# Development and application of an indicator assessment tool for measuring health services accreditation programs

**DOI:** 10.1186/s13104-015-1330-6

**Published:** 2015-08-20

**Authors:** Virginia Mumford, David Greenfield, Anne Hogden, Deborah Debono, Kevin Forde, Johanna Westbrook, Jeffrey Braithwaite

**Affiliations:** Centre for Clinical Governance Research, Australian Institute of Health Innovation, UNSW, Sydney, Australia; School of Public Health and Community Medicine, UNSW, Sydney, Australia; Centre for Health Systems and Safety Research, Australian Institute of Health Innovation, UNSW, Sydney, Australia

**Keywords:** Quality indicators, Healthcare acquired infection, Accreditation, Hand hygiene

## Abstract

**Background:**

Hospital accreditation programs are internationally widespread and consume increasingly scarce health resources. However, we lack tools to consistently identify suitable indicators to assess and monitor accreditation outcomes. We describe the development and validation of such a tool.

**Results:**

Using Australian accreditation standards as our reference point we: reviewed the research evidence for potential indicators; looked for links with existing external indicators; and assessed relevant state and federal policies. We allocated provisional scores, on a five point Likert scale, to the five accountability criteria in the tool: research; accuracy; proximity; no adverse effects; and specificity. An expert panel validated the use of the purpose designed indicator assessment tool. The panel identified hand hygiene compliance rates as a suitable process indicator, and hospital acquired *Staphylococcus aureus* infection (SAB) rates as an outcome indicator, with the hypothesis that improved hand hygiene compliance rates and lower SAB rates would correlate with accreditation performance.

**Conclusions:**

This new tool can be used to identify, analyse, and compare accreditation indicators. Using infection control indicators such as hand hygiene compliance and SAB rates to measure accreditation effectiveness has merit, and their efficacy can be determined by comparing accreditation scores with indicator outcomes. To verify the tool as a robust instrument, testing is needed in other health service domains, both in Australia and internationally. This tool provides health policy makers with an important means for assessing the accreditation programs which form a critical part of the national patient safety and quality framework.

## Background

Health services accreditation programs are designed to strengthen quality and safety improvement efforts through compliance with clinical and organisational standards. Accreditation programs have been widely adopted with over 27 country specific hospital accreditation agencies active in a 2009 survey [1], and several international agencies providing cross-border accreditation services. However, the evidence for accreditation improving patient safety and quality is mixed [2–4]. An Australian study showed accreditation improved organisational measures [5], and a European study indicated that external assessment systems are associated with better clinical outcomes [6]. Other work has shown a transient effect [7], or no effect on clinical outcomes [8]. One of the difficulties in assessing the research and the range of findings is the lack of a comprehensive framework by which to analyse and compare the costs and benefits of accreditation programs. To meet this need and as part of the Accreditation Collaborative for the Conduct of Research, Evaluation and Designated Investigations through Teamwork (ACCREDIT) research partnership [9–11], we established a framework for evaluating the costs and benefits of hospital accreditation [12]. Our purpose designed SIQNS framework comprises five discrete activities: (1) **S**cope and objectives; (2) **I**dentify costs and benefits; (3) **Q**uantify costs and benefits; (4) calculate **N**et social benefits; and (5) **S**ensitivity analysis.

There are a number of indicators that have been validated and accepted for measuring patient safety and quality in healthcare [13]. However, one of the additional challenges in measuring the effectiveness of accreditation is in identifying indicators for the outcomes or benefits that can be linked specifically to the accreditation process. We therefore developed an indicator assessment tool to identify, compare and evaluate the suitability of either process or outcome indicators in hospital accreditation as part of our SIQNS framework. This tool incorporated the four accountability criteria used by The Joint Commission (the main United States health services accreditation agency) to measure improvements in the clinical processes of care [14]. These were: level of research evidence underpinning the indicator; accuracy of the indicator in measuring implementation of the standard; proximity of the standard and indicator; and the potential for adverse effects or unintended consequences. We adapted these criteria to assess accreditation and added an additional criterion, specificity, to account for the difficulties of isolating the effects of accreditation from other quality and safety interventions (Table 1).

**Table 1:** Indicator assessment tool—criteria explanation

Indicator assessment criteria	Explanation
Research	What is the evidence that compliance with the accreditation standard affects the indicator?
Accuracy	How accurate is the indicator in terms of measuring compliance with the accreditation standard?
Proximity	How close is the link between the standard and the indicator? Is there a causal chain?
No adverse effects	What is the risk of avoiding adverse effects?
Specificity	Is it possible to isolate the effects of accreditation on the indicator from other safety and quality programs?

The aim of this study is to describe the development and validation of a purpose designed indicator assessment tool in identifying indicators for hospital accreditation performance. An expert panel assessed the tool using hand hygiene compliance rates and *Staphylococcus aureus* (SAB) rates to illustrate the selection process and assess the suitability of these indicators in assessing the impact of accreditation on patient safety and quality of care.

## Research methods

### Study setting and process

This study takes the health service accreditation standards for Australian hospitals as its reference point. We reviewed the ten National Safety and Quality Health Service (NSQHS) Standards developed by the Australian Commission on Safety and Quality in Health Care (ACSQHC) and introduced in early 2013 [15]. In addition, we reviewed the accreditation standards within the Evaluation and Quality Improvement Program (EQuIP), issued and maintained by the Australian Council for Healthcare Standards (ACHS) [16, 17]. We convened an expert panel to assess the tool, using two key indicators to demonstrate the selection process.

### Indicator selection

We focused on the first three NSQHS standards: Standard 1 *Governance for safety and quality in health service organisations*; Standard 2 *Partnering with consumers*; and Standard 3 *Preventing and controlling healthcare associated infection*s [15]. For each standard we reviewed the research evidence; looked for links with existing external indicators; and reviewed relevant state and federal policies in Australia. Given the recent introduction of the NSQHS Standards we also checked that the pre-existing EQuIP accreditation standards contained relevant content for retrospectively comparing indicators with accreditation outcomes [16, 17].

### Evaluation of accreditation indicators

Four researchers from the ACCREDIT team assessed potential indicators against the five criteria in our indicator assessment tool during a series of four evaluation meetings. The researchers were experienced in a wide range of clinical and health services research expertise including: qualitative and quantitative methods; patient safety and quality; and organisational culture. Each criterion was allocated a score ranging from low to high on a five point Likert scale (Table 2). For example, high scores were allocated to the research criterion for evidence based on a systematic review, or acceptance by a national or international health body. A low score in research would be allocated where evidence was weak, for example from individual case studies. Since the criteria are measuring different aspects of each standard, these scores were considered separately and not used to create an overall score.

**Table 2:** Indicator assessment tool—score allocation examples

Indicator assessment criteria	Scoring system for indicator assessment tool	
	Low score	High score
Research	Low level of evidence. For example, results of individual case studies	High level of evidence. For example systematic reviews, or adoption by regional or national health bodies
Accuracy	The indicator only measures a small part of the standards, or is not sufficiently or independently verified	The indicator measures whether the accreditation intervention has been implemented or the service provided in accordance with the standard
Proximity	Multiple steps or processes between the indicator measurement and accreditation standard make it difficult to link the indicator with the effects of accreditation	Close temporal or process links exist between accreditation surveys and the indicator measurement
No adverse effects	Measurement or publication of results gives incentives for gaming or misallocation of resources	Indicator measurement or publication does not give rise to unintended adverse effects or consequences
Specificity	Multiple and recent policy initiatives covering the same topic as the accreditation standard under discussion	Accreditation is the main policy implementation tool

### Review and assessment of indicator tool

We next convened an expert panel to validate the results of our prototype indicator assessment tool building on methods adopted for assessing quality indicators in primary health care [18]. This panel comprised seven experienced, Australian based researchers working on accreditation and quality outcomes, as well as three high profile international healthcare quality and safety experts from Spain and the United Kingdom. The panel session comprised a 2 h meeting with all members present in person. Our panel members averaged 12.7 years in health services research and a total authorship of more than 1000 health research publications. We presented the panel with a description of the tool, the indicative scores allocated by the ACCREDIT team, the rationale for those scores, and the research evidence for the indicators. We used an evidence-based consensus approach to synthesise the results of the panel [18]. The tool was additionally endorsed by an ACCREDIT Steering Committee comprising accreditation agency senior executives, health quality bodies, and health service managers.

## Results

### Indicator selection

We focused on Standard 3 relating to hospital acquired infection. This was partly due to the specificity of the interventions that could be mapped across the ACHS and NSQHS Standards. In addition, infection control processes have become increasingly important as infections acquired during a hospital stay are a major cause of morbidity and result in significant costs in terms of increased length of stay and additional treatment [19]. Standard 3 comprises six main topics: governance and systems; patient management; infection prevention and control strategies; antimicrobial stewardship; cleaning, disinfecting and sterilisation; and communicating with patients and carers [20]. Hand hygiene compliance rates and *Staphylococcus aureus* bacteraemia (SAB) incidence rates in the Australian acute care sector are routinely collected and published, and we selected these indicators to test our indicator assessment tool.

Hand hygiene programs in Australian hospitals, based on World Health Organization recommendations [21], take a multimodal approach, and are in line with best practice internationally [22]. This approach incorporates a number of organisational and safety components including: staff education, appropriate infrastructure; compliance monitoring; and communicating with patients. This broad approach to quality improvement reflects the goals of the accreditation program within an overarching safety and quality framework [6].

The ACSQHC has recommended that hand hygiene programs in Australia are repeatedly monitored using both process indicators (compliance rates) and outcome indicators (infection rates) [23]. Auditors trained by Hand Hygiene Australia (the agency tasked by ACSQHC to implement the program) monitor hand hygiene activity against the total number of potential “moments” for hand hygiene. These five “moments” comprise: before touching a patient; before a procedure; after a procedure or body fluid exposure risk; after touching a patient; and after touching a patient’s surroundings [24]. The national target is a compliance rate of 70 % and results are publicly reported three times a year, providing readily accessible indicator data [24]. SAB rates are reported on a monthly basis, with a national target incidence rates of <2 per 10,000 inpatient bed days [25].

### Indicator evaluation

The panel collectively reviewed the five criteria, assessed the evidence and interim evaluation provided, and awarded each a score ranging from low to high. The scores varied, with higher scores for research and proximity to lower scores for specificity. The results and rationale for the allocated scores for hand hygiene compliance rates are shown in Table 3, and those for SAB rates are shown in Table 4.

**Table 3:** Expert panel’s evaluation of hand hygiene compliance as a process indicator

Topic	Infection control indicators
*Indicator details*	
Description	Audits of compliance with the “Five Moments” of hand hygiene
Type	Process
*Applicable accreditation standards*	
NSQHSS	Standard 3—Preventing and controlling healthcare associated infections [15]
ACHS EQuIP	Criteria 1.5.2 [16, 17]

	Scores	Evaluation discussion summary
*Assessment criteria*		
Research	High	This was given a high score due to the evidence linking hand hygiene outcomes with the multimodal approach used in Australian accreditation programs as per WHO guidelines [21–24, 26–30]
Accuracy	Medium to low	Hand hygiene audits were seen as an accurate method for measuring compliance with the sections of the accreditation standard dealing with preventing and controlling infections. However, a medium to low score was allocated due to the broad nature of the infection control standard that also includes anti-microbial stewardship and specialist cleaning policies
Proximity	High	Audits are performed three times a year, creating a close temporal link between hand hygiene audits and accreditation surveys. There is an organizational link between the accreditation criteria and the requirements for implementing hand hygiene programs
No adverse effects	Medium	The data are self-audited against a known national target providing incentives for hospitals to meet those targets, and some potential for gaming
Specificity	Low	There are a number of policies and initiatives aimed at improving hand hygiene rates making it difficult to isolate the effects of accreditation. For example, both the Clean Hands Save Lives project introduced by the NSW Clinical Excellence Commission and the National Hand Hygiene Initiative contained elements of the accreditation standards relating to hand hygiene and infection control
Associated programs	Related programs initiated by state or federal governments or healthcare agencies	Anti-microbial Stewardship Initiative [31] National Infection Control Guidelines [32] Clean Hands Save Lives project [33] National Hand Hygiene Initiative [24] NSW Infection Control Policy [34]

**Table 4:** Expert panel’s evaluation of SAB rates as an outcome indicator

Topic	Infection control indicators
*Indicator details*	
Description	Incidence of hospital acquired *Staphylococcus aureus* bacteraemia (SAB rates) per 10,000 inpatient days. Rates are reported monthly for publicly funded acute care hospitals
Type	Outcome
*Applicable accreditation standards*	
NSQHSS	Standard 3—preventing and controlling healthcare associated infections [15]
ACHS EQuIP	Criteria 1.5.2 [16, 17]

	Scores	Evaluation discussion summary
*Assessment criteria*		
Research	Medium to high	There is extensive research on hand hygiene and other infection control mechanisms in reducing infection rates. [21, 22, 26, 27, 29] However, there is little evidence that the process of accreditation has an impact on infection rates
Accuracy	Low to medium	A medium to low score was allocated due to the broad nature of the infection control standard that also includes anti-microbial stewardship and specialist cleaning policies
Proximity	High	Results are collected monthly as part of routine reporting
No adverse effects	High	Results are publicly available with a known target incidence rate but are routinely collected and can be corroborated with pathology lab reports
Specificity	Low	There are a number of policies and initiatives aimed at improving hospital acquired infection (see associated programmes below)
Associated programs	Related programs initiated by state or federal governments or healthcare agencies	Anti-microbial Stewardship Initiative [31] National Infection Control Guidelines [32] Clean Hands Save Lives project [33] National Hand Hygiene Initiative [24] NSW Infection Control Policy [34]

### Tool and indicator validation

The expert panel endorsed the indicator assessment tool and its suitability for assessing process and outcome indicators for accreditation. The panel determined that hand hygiene would be suitable as a process indicator for hospital accreditation and should be assessed against accreditation outcomes. The higher scores for research and proximity were critical in this consideration and provide both face and content validity for the tool [18]. The panel also recommended investigating SAB rates as an outcome indicator based on higher scores for research, proximity, and no adverse effect. The lower scores for specificity and accuracy were noted for both of these indicators. The panel recommended using the systematic approach embedded within the tool to widen the topics covered and include a range of indicators from across the accreditation standards.

## Discussion and conclusions

Our validated indicator assessment tool can be used to systematically identify process and outcome indicators in order to assess their suitability as indicators of accreditation performance in Australian hospitals [35]. This study focused on the infection control standard to illustrate the application of the tool. Further validation with a broader range of healthcare workers and across all ten NSQHS standards is merited. Identifying indicators of accreditation performance provides health policy makers with an important means for assessing a critical part of the patient safety and quality framework, both for monitoring the effect of current accreditation programs and in assessing their development. Testing is needed to ensure applicability of the tool in international and domestic accreditation programs across the acute, primary and aged care health domains.

## Abbreviations

ACCREDIT: accreditation collaborative for the conduct of research, evaluation and designated investigations through teamwork
ACHS: Australian Council of Healthcare Standards
ACSQHC: Australian Commission on Safety and Quality in Health Care
EQuIP: Evaluation and Quality Improvement Program NSQHS standards: national safety and quality health service standards
NSW: new south wales
SIQNS: a framework for evaluating the costs and benefits of health services accreditation (scope, identify, quantify, net social benefit calculation, and sensitivity)
WHO: World Health Organization
